# Quantum gravity: are we there yet?

**DOI:** 10.1098/rsta.2023.0377

**Published:** 2025-05-08

**Authors:** Shahn Majid

**Affiliations:** ^1^ School of Mathematical Sciences, Queen Mary University of London, London, UK

**Keywords:** non-commutative geometry, quantum groups, quantum gravity, quantum spacetime

## Abstract

The turn of the millennium was a time of optimism about an approach to non-commutative geometry inspired by rich mathematical objects called ‘quantum groups’ and its applications to quantum spacetime. The idea was to model quantum gravity effects as non-commutativity of spacetime coordinates and arguably solve quantum gravity itself. This required, however, a 20-year development of a suitable formalism of *quantum Riemannian geometry* (QRG) to handle such coordinates, which I outline here. I then provide new results for state-of-the-art fuzzy sphere and n-gon ‘baby quantum gravity’ models in this approach and some possible features of quantum gravity that these models suggest. Also discussed are the critical conceptual and mathematical elements that are still missing to more fully achieve this goal.

This article is part of the theme issue ‘Science into the next millennium: 25 years on’.

## 1. Introduction

While the start of the twentieth century told us that position and momentum coordinates of mechanical systems are better modelled by quantum theory as operators that do not commute (the famous Heisenberg uncertainty relations), the tail end of the twentieth century saw an emergence of the view that spacetime coordinates might need to not commute, this time due to quantum gravity corrections to the geometry. This is the *Quantum Spacetime Hypothesis* and is one of the first (and still the most widely studied) models of it type, the bicrossproduct model spacetime [1]:


(1.1) $$\begin{array}{lcr} & [x^{i},t]=ı\lambda_{P}x^{i},\;[x^{i},x^{j}]=0, & \end{array}$$

where $x^{i}=x,y,z$ are spatial coordinates, t is time and $\lambda_{P}∼10^{-44}$ s is the Planck time, an extremely small constant. The idea is that there is lots of evidence that spacetime at this sort of timescale or, equivalently, at the Planck length scale $10^{-35}$ m, is not a continuum but something else, albeit a deep mystery in the absence of a theory of quantum gravity. The quantum spacetime hypothesis is a concrete proposal for how that ‘something else’ could be better modelled, even without knowing quantum gravity, and hence is a better foundation on which to build.

One might complain at this point that surely a model such as equation (1.1) depends on the reference frame, i.e. it breaks Einstein’s principle of special relativity. Actually what happens is that the entire Poincaré group of translations and Lorentz transformations are also modified, using the notion of a *quantum group.* This was a generalisation of group theory that emerged in the 1980s (although the axioms, but without convincing examples, were proposed by the mathematician H. Hopf in the 1940s). One class of these, the q-deformations [2], came out of integrable systems, and another class, the bicrossproducts [3], came out of the search for models of quantum gravity. The relevant quantum group $C(H^{1,3})\;▸\;\;◃U(so_{1,3})$ for the above model is one of the latter class, albeit a quantum group isomorphic to it (without an action on a quantum spacetime) was first proposed in [4] as a contraction limit of $U_{q}(so_{2,3})$, i.e. the two are not unconnected. One might also complain that as $\lambda_{P}$ is so small, isn’t this model totally untestable? However, there are situations where quantum-gravity effects can be magnified and (in principle) be measurable. For example, a predicted small energy dependence on the speed of light in the above model would result in different arrival times of a few milliseconds for γ-ray bursts of cosmological origin [5]. Another effect could be a phase transition such as ‘Bose–Einstein condensation’ (albeit, so far modelled in mathematically analogous situations rather than quantum gravity itself). There have also been models proposed on other theoretical grounds, such as in string theory as an effective model of the motion of the ends of open strings landing on a d-brane [6], or a model in [7] keeping Lorentz symmetry. A concrete model has also emerged in Euclideanised 2 + 1 quantum gravity, which is exactly solvable but topological (i.e. without dynamics of the graviton), known as the ‘fuzzy ${\mathbb{R}}^{3}$’ model:


(1.2) $$[x^{i},x^{j}]=2ı\lambda_{P}\epsilon_{ijk}x^{k},$$

where $\lambda_{P}$ is of the order of the Planck length and $\epsilon_{123}=1$ is extended as a totally antisymmetric tensor. This was proposed in [8], but the reader may be familiar with it in another context as the algebra of angular momentum, i.e. the enveloping algebra $U(su_{2})$. This fuzzy ${\mathbb{R}}^{3}$ has a quantum Poincaré group $C(SU_{2})⋊U(su_{2})$ as a special case (a ‘quantum double’) of a bicrossproduct [9,10].

If such models of flat but quantum spacetime were the state of the art around the turn of the millennium, where did they need to go next, and where did they go next? The bottom line is that while they motivated speculative ideas for ‘quantum gravity phenomenology’, they still needed two fundamental issues to be addressed:

(i) How to extend such models to quantum but curved spacetimes such as around a black hole or in cosmological models
(ii) How to extract physical predictions from the mathematics in a coordinate-free way

Neither is an easy task and it has taken the next 20 years, i.e. up until now, to address them in a practical manner. On the first problem, powerful approaches to non-commutative geometry already existed, notably those of Alain Connes [11,12] who, among other things, encoded geometric information in spectral data (notably via an abstract ‘Dirac operator’). But this did not give direct access to analogues of familiar geometric objects such as the metric tensor and was also not typically well adapted to examples coming from quantum groups. Instead, what emerged was a ‘layer-by-layer’ approach dubbed *quantum Riemannian geometry* (QRG), in which we start with a potentially non-commutative coordinate algebra A, choose its differential structure in the sense of a bimodule of 1-forms $\Omega^{1}$, choose a metric $g\in \Omega^{1}{⊗}_{A}\Omega^{1}$ and solve for a *quantum Levi-Civita connection* (QLC) $\nabla :\Omega^{1}\to \Omega^{1}{⊗}_{A}\Omega^{1}$, with Riemann curvature $R_{\nabla }$. This is now covered in my text with Beggs [13] building on key works such as [14–17]. The state of the art here is that while $R_{\nabla }$ is canonical, there is only a ‘working definition’ of the Ricci curvature. But this is enough to build the first ‘baby quantum gravity’ models [18–21] and get a first look at quantum gravity.

The second problem is equally fundamental. In General Relativity, calculations can be done in any coordinate system, but it is then very hard to know what aspects of what one computes are due to the choice of coordinates and what are actually physical, independent of the coordinates. Key here is the notion of a geodesic as a way to map out the geometry of a continuum spacetime in a coordinate-invariant way by looking at how particles move in it. The same issues arise for quantum spacetime not only on the geometric side but even in flat spacetime, i.e. working with specific generators and relations, how can we extract the actual physics? This was recently addressed in the notion of ‘quantum geodesics’ [22–25].

Before we begin, here is a current wish-list of things upon which we might hope quantum gravity could shed light.

(1) What happens at the centre of a black hole or at the initial ‘Big Bang’ where, classically, the curvature diverges?
(2) Can we explain the problem of the ‘cosmological constant’? To match observed cosmology to Einsteins gravity, one needs to add an extra cosmological constant term or, equivalently, an unexplained ‘dark energy’ to the stress tensor of a vacuum. The energy density required is of order $10^{-29}$g cm^-3^ and the problem is why, if it is a quantum gravity effect, is it so small compared to the Planck density and yet non-zero?
(3) Can we more deeply understand an apparent link between curvature and entropy as suggested by Bekenstein–Hawking radiation near a black-hole?
(4) Can we understand the Diosi–Penrose idea of ‘gravitational state reduction’ where quantum systems are proposed to be spontaneously measured due to interaction with gravity?
(5) Can we understand the structure of the Standard Model, i.e. the particular elementary particles observed in accelerators, and their particular masses, as emerging from quantum gravity?

There is too little space here to discuss these points in any depth or to review or connect with other approaches to quantum gravity. Instead we focus on the QRG approach. We first provide a brief outline of the QRG formalism, which readers who do not like algebra should skip. Section 3 contains the new results of the paper, that is, an in-depth further study of two baby quantum gravity models, not published elsewhere. In §§4 and 5 we cover some other key topics, and conclude in §6 with a summary of how far along we are on the wish-list and what are the main obstructions we need next to overcome.

## 2. QRG formalism

A lightning introduction to the formalism is as follows.

(i) We pick a ∗-algebra A in the role of complexified coordinates on a continuum manifold, i.e. equipped with a conjugate linear map ∗:A→A reversing products and squaring to the identity. In the commutative case, one could look at the real subalgebra of self-adjoint elements (which in the classical case would be the usual real coordinate algebra) but in the non-commutative case one just remembers ∗ as specifying the ‘real form’. In quantum mechanics, A could be a matrix algebra and then ∗ is just hermitian conjugation.
(ii) We pick a graded algebra $\Omega ={⊕︎}_{i}\Omega^{i}$ in the role of differential forms of various degrees i. This replaces ‘differential calculus’ in classical geometry and as such is equipped with $d:\Omega^{i}\to \Omega^{i+1}$ obeying a graded-Leibniz rule. We assume Ω is generated by $\Omega^{0}=A$ and $\Omega^{1}=AdA$ and that ∗ extends to Ω as a graded-involution commuting with d. The product of differential forms is denoted ∧.
(iii) We pick a quantum metric $g\in \Omega^{1}{⊗}_{A}\Omega^{1}$. Although geometers do not think algebraically, this is actually what they are doing when they write a metric as $g=g_{\mu \nu }dx^{\mu }dx^{\nu }$ in local ordinates on a manifold M, where $\left\{dx^{\mu }\right\}$ are a basis of 1-forms and there is a hidden ${⊗}_{C^{\infty }(M)}$ in the middle of $dx^{\mu }dx^{\nu }$ to be understood. We need g to be invertible in the sense of a map $(,):\Omega^{1}{⊗}_{A}\Omega^{1}\to A$ which classically would be given by the inverse metric tensor $g^{\mu \nu }=\left(dx^{\mu },dx^{\nu }\right)$. (In mathematical terms, $\Omega^{1}$ is an A-bimodule and a quantum metric makes it isomorphic to its dual in the monoidal category of A-bimodules.) We usually impose some form of ‘quantum symmetry’ in addition, such as ∧(g)=0. We also require ‘reality’ in the form

†(g)=g,†=flip(∗⊗∗),

which in local coordinates in the classical case would ensure that the coefficients in a self-adjoint basis are real.

(iv) A Riemannian connection is formulated as a map $\nabla :\Omega^{1}\to \Omega^{1}{⊗}_{A}\Omega^{1}$ with certain properties. Note that a ‘right vector field’ is a map $X:\Omega^{1}\to A$ which respects right-multiplication by A and in this case $\nabla_{X}=(X⊗\mathrm{id})\nabla$ is the associated ‘covariant derivative’ familiar in the continuum case. The connection map is required to obey

∇(aω)=da⊗ω+a∇ω,∇(ωa)=σ(ω⊗da)+(∇ω)a

for all $a\in A,\omega \in \Omega^{1}$*,* for some bimodule map $\sigma :\Omega^{1}{⊗}_{A}\Omega^{1}\to \Omega^{1}{⊗}_{A}\Omega^{1}$ which we assume invertible. The latter in classical geometry would be an invisible ‘flip’ of indices needed for a vector field X to couple correctly on the left-most copy of $\Omega^{1}$. It is not additional data but, if it exists, determined by ∇ via the above. The category of such ‘bimodule connections’ is closed under tensor product, so ∇ extends to a connection on $g\in \Omega^{1}{⊗}_{A}\Omega^{1}$ and we say it is *metric compatible* if ∇(g)=0. There is also a torsion tensor of any connection:


$$T_{\nabla }=\wedge \nabla -d:\Omega^{1}\to \Omega^{2},$$

comparing the exterior derivative $d:\Omega^{1}\to \Omega^{2}$ with ∇ followed by ∧. A *quantum Levi–Civita connection* (QLC) is defined as a ∇ which is metric compatible and has $T_{\nabla }=0$. Unlike in classical geometry, it need not exist and even if it does, it need not be uniquely determined by g. This means that not every classical metric is quantizable to QRG as we currently formulate it, which we see as a good thing as it may single out particular classical metrics as arising from QRG (see §4). We also require reality or ∇∗*-preserving* in the sense


$$\sigma^{-1}\circ \nabla \circ *=†\nabla .$$

In the classical case, this ensures that the Christoffel symbols or connection coefficients with respect to a self-adjoint basis are real.

(v) The Riemann curvature of a connection is a map:

$$R_{\nabla }=(d⊗\mathrm{id}-\mathrm{id}\wedge \nabla )\nabla :\Omega^{1}\to \Omega^{2}{⊗}_{A}\Omega^{1}.$$

Both this and $T_{\nabla }$ are well-defined for any left connection, i.e. not using the Leibniz rule involving σ. This ‘algebraic’ formulation of geometry will be very alien to readers more familiar with General Relativity, where formulae are generally given in local coordinates and tensor calculus, but in many ways is cleaner and more conceptual.

The Ricci tensor is less clear and needs some kind of interior product $\Omega^{1}{⊗}_{A}\Omega^{2}\to \Omega^{1}$ to be able to trace $R_{\nabla }$. The simplest additional data here are to suppose a bimodule lifting map $i:\Omega^{2}\to \Omega^{1}{⊗}_{A}\Omega^{1}$, which in classical geometry simply writes a 2-form as an antisymmetric combination of 1-forms (i.e. with two, antisymmetric, indices). In QRG, we specify it so that ∧∘i=id and ideally i∘∗=−∗∘i, as this would be true in classical geometry. Then,


$$\mathrm{Ricci}=((\;,\;)⊗\mathrm{id})(\mathrm{id}⊗i⊗\mathrm{id})(\mathrm{id}⊗R_{\nabla })g\in \Omega^{1}{⊗}_{A}\Omega^{1},$$

after which we define the *Ricci scalar curvature:*


R=(,)Ricci.

This is needed for the action for quantum gravity. But note that the natural QRG Ricci *reduces in the classical case to* $-\frac{1}{2}$ *of the usual value.* There is also a natural QRG Laplacian Δ=(,)∇d on A which provides the action for a scalar field on the QRG.

## 3. Baby quantum gravity models

To write down quantum gravity on a QRG, we need two more things. There is usually a natural choice for each of these but no fully general theory, i.e. they have to be chosen for each model. One is a notion of integration ∫:A→ℂ which sends non-zero positive elements to ${\mathbb{R}}_{>0}$. The second is, even if we can solve for the moduli $M_{QRG}$ of QRG’s for a given differential algebra $\left(A,\Omega^{1},\Omega^{2},d\right)$*,* we need to choose a measure $\mu_{g}$ for integration over this classical moduli space. Then we can write the quantum gravity partition function


(3.1) $$Z=\int_{M_{QRG}}dg\;\mu_{g}\;e^{\frac{ı}{G}\int R},$$

where G is a real coupling (say, positive) constant. We are suppressing here that there may also be parameters in the QLC (it need not be unique in QRG), and these should also be either fixed as background data or integrated over. For Euclideanised quantum gravity, we replace the imaginary unit ı in the exponent by ±1 according to which way ∫R is bounded.

### (a) Quantum gravity on the fuzzy sphere revisited

The unit fuzzy sphere has self-adjoint generators $x^{i}$ with relations


$$[x^{i},x^{j}]=2ı\lambda_{P}\epsilon_{ijk}x^{k},\;\sum_{i}(x^{i}{)}^{2}=1-\lambda_{P}^{2},$$

where $\lambda_{P}$ is a dimensionless parameter in the role of Planck length, except that we divided through by the size of the sphere (one could scale the $x^{i}$ and $\lambda_{P}$ by this to work with physical dimensions). Aside from this, we see the fuzzy ${\mathbb{R}}^{3}$ model (equation (1.2)) with the quadratic relation of a sphere imposed. Conventions are chosen so that if $\lambda_{P}=\frac{1}{n}$ for a natural number n, the algebra has a finite-dimensional representation in n×n matrices. Adding further relations in this case gives something equivalent to working with the algebra of n×n matrices, which are also called fuzzy spheres for each n in the literature [26]. Next, there is a natural differential calculus [13] with three self-adjoint central basis 1-forms $s^{i}$. These correspond classically to the Killing forms for the action of $SU_{2}$, under which the fuzzy sphere and calculus are covariant (classically, these do not form a basis but this is not the case for $\lambda_{P}\neq 0$). A metric is therefore of the form,


$$g=g_{ij}s^{i}⊗s^{j},$$

for a positive real symmetric matrix $\left\{g_{ij}\right\}$. The Ricci scalar works out [19] as


$$R=\frac{1}{2\;\det(g)}\left(\mathrm{Tr}(g^{2})-\frac{1}{2}\mathrm{Tr}(g{)}^{2}\right).$$

We proceed as in [19] with integration measure ∫1=det⁡(g) for the action and with a spectral decomposition of the metric in terms of eigenvalues $\lambda_{1},\lambda_{2},\lambda_{3}>0$ and Euler angles. In these terms, the Ricci scalar is


$$R=\frac{1}{4\lambda_{1}\lambda_{2}\lambda_{3}}\left(\lambda_{1}^{2}+\lambda_{2}^{2}+\lambda_{3}^{2}-2(\lambda_{1}\lambda_{2}+\lambda_{2}\lambda_{3}+\lambda_{3}\lambda_{1})\right).$$

If we are only interested in observables which are functions of the eigenvalues, then the effective partition function for Euclideanised quantum gravity is


(3.2) $$Z=\int_{\epsilon }^{L}\prod_{i}d\lambda_{i}\;\frac{|(\lambda_{1}-\lambda_{2})(\lambda_{2}-\lambda_{3})(\lambda_{3}-\lambda_{1})|}{\lambda_{1}^{2}\lambda_{2}^{2}\lambda_{3}^{2}}\;e^{-\frac{1}{2G^{2}}(\lambda_{1}^{2}+\lambda_{2}^{2}+\lambda_{3}^{2}-2(\lambda_{1}\lambda_{2}+\lambda_{2}\lambda_{3}+\lambda_{3}\lambda_{1}))},$$

where we cut-off at both ends to $\epsilon <\lambda_{i}<L$, to control divergences. The physical values of L,ϵ,G have dimensions length^2^, and it is for this reason that we squared G compared to [19] and the general scheme. The physical roles of L,ϵ are metric field strength cut-offs. So if L→∞ dominates then it means that large macroscopic geometries contribute most to the quantum gravity calculation, while if ϵ→0 dominates then small geometries contribute most. There is no exact comparison with regular quantum field theory but one can roughly think of these as like ‘infrared’ and ‘ultraviolet’ cut-offs, respectively.

Henceforth, we set G=1, either because we are working in Planckian units or because we can just use rescaled variables and parameters


$$\lambda_{i}=\frac{\lambda_{i}^{phys}}{G},\;L=\frac{L^{phys}}{G},\;\epsilon =\frac{\epsilon^{phys}}{G},$$

where $\lambda_{i}^{phys},L^{phys},\epsilon^{phys}$ are what we previously called $\lambda_{i},L,\epsilon$. In terms of the new dimensionless variables, Z looks identical, but with G=1.

### (b) Higher-order corrections for large L

**Figure 1.**
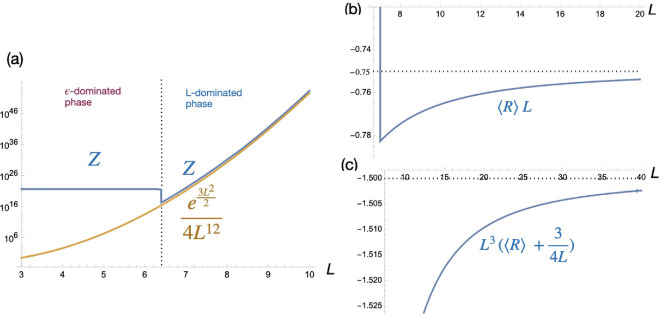
(a) Numerical behaviour of partition function Z for quantum gravity on the fuzzy sphere as a function of cut-off L. (b) Expectation value of Ricci scalar showing rapid convergence of ⟨R⟩L to −3/4 and (c) first quantum gravity correction. Plots are for $\epsilon =10^{-20}$.

Our first new result is that


$$Z∼\frac{1}{4L^{12}}e^{\frac{3L^{2}}{2}},$$

for L≫6, with increasing accuracy as L→∞. This is a very good approximation for L>35 and also matches well in the log-plot figure 1a. The value of ϵ is not significant in this regime; we use $\epsilon =10^{-20}$ as default in this section, but plots for large L are not visibly different all the way up to $\epsilon =10^{-1}$. We also see very different behaviour for L≪6 to be discussed later.

Next, for expectation values, we insert an operator into the integral for Z and divide the result by Z. Then for large L, one has


$$\langle \lambda_{i_{1}}\cdots \lambda_{i_{n}}\rangle ∼L^{n}$$

as L→∞, at least for positive powers. This is a correction to [19], where there was a constant coefficient $\frac{3}{16}$ due to a coding error in the calculation. Our new result here is that to leading order


(3.3) $$\begin{array}{lcr} & \Delta \lambda_{i}:=\sqrt{\langle \lambda_{i}^{2}\rangle -\langle \lambda_{i}\rangle^{2}}∼\sqrt{\frac{41}{12}}\frac{1}{L} & \end{array}$$

as L→∞, as can be deduced from the more detailed expansion


$$\langle \lambda_{i}\rangle =L-\frac{25}{6L}-\frac{1}{18L^{3}}+O(\frac{1}{L^{4}}),\;\langle \lambda_{i}\lambda_{j}\rangle =\left\{ \begin{array}{ll} L^{2}-\frac{17}{3}+\frac{7}{3L^{2}}+O(\frac{1}{L^{3}}) & i\neq j,\\ L^{2}-\frac{25}{3}+\frac{62}{3L^{2}}+O(\frac{1}{L^{3}}) & i=j. \end{array}\right.$$

Here, equation (3.3) that the theory does exhibit quantum gravity (there is quantum uncertainty in the metric values) but less so at large L, which makes sense as this would be more dominated by macroscopic behaviour. Similarly, one has


(3.4) $$\langle R\rangle =-\frac{3}{4L}-\frac{3}{2L^{3}}+O(\frac{1}{L^{4}}),$$

as shown in figure 1b,c. The leading term here is (in QRG conventions) the Ricci curvature of a unit fuzzy sphere with metric $g_{ij}=L\delta_{ij}$, in other words the unquantized or macroscopic value. The next term is then the leading quantum gravity correction. Also visible in figure 1b is that ⟨R⟩L, like Z, suddenly jumps to a very large positive value as L goes below a critical value.

### (c) Renormalization of the gravitational coupling constant G

We can translate these results back to the physical values defined via $\lambda_{i}^{phys}$. The Ricci curvature computed from the latter is


$$R^{phys}=\frac{R}{G},\;\langle R^{phys}\rangle =\frac{\left\langle R\right\rangle }{G}∼-\frac{3}{4\langle \lambda_{i}\rangle G}=-\frac{3}{4\langle \lambda_{i}^{phys}\rangle }$$

for L→∞. We do not need it if we only look at ratios of physical quantities, but we can also consider G=G(L) and choose this so that


$$\langle \lambda_{i}^{phys}\rangle :=G(L_{0})\langle \lambda_{i}\rangle_{L_{0}}$$

is fixed as some arbitrary value at some $L_{0}$ of the regularization parameter L. The subscript is used to indicate our above computations done at $L_{0}$. Then one can in principle solve for G(L) in such a way that


$$\langle \lambda_{i}^{phys}\rangle =G(L)\langle \lambda_{i}\rangle_{L},$$

i.e. the right-hand side is constant for all L. This gives


$$G(L)=\frac{\left\langle \lambda_{i}^{phys}\right\rangle }{\langle \lambda_{i}\rangle_{L}}=G(L_{0})\frac{\langle \lambda_{i}\rangle_{L_{0}}}{\langle \lambda_{i}\rangle_{L}}∼\frac{G(L_{0})L_{0}}{L}(1-\frac{25}{6L_{0}^{2}})(1+\frac{25}{6L^{2}})$$

for large $L_{0},L$ using our above results. Using more precise information about $\langle \lambda_{i}\rangle_{L}$, one can get more precise information on G(L). We can then compute


$$\langle R^{phys}\rangle_{L}=\frac{\langle R\rangle_{L}}{G(L)}=\langle R\rangle_{L}\frac{\langle \lambda_{i}\rangle_{L}}{\left\langle \lambda_{i}^{phys}\right\rangle }∼\left(-\frac{3}{4}+\frac{13}{8L^{2}}+O(\frac{1}{L^{3}})\right)\frac{1}{\left\langle \lambda_{i}^{phys}\right\rangle }$$

for large L. The value here depends on the regulator L but to the lowest order in large L, we can replace this by $\langle \lambda_{i}^{phys}\rangle /G$ so that in physical terms


$$\langle R^{phys}\rangle ∼\left(-\frac{3}{4}+\frac{13G^{2}}{8\langle \lambda_{i}^{phys}\rangle^{2}}\right)\frac{1}{\left\langle \lambda_{i}^{phys}\right\rangle }$$

plus higher-order corrections. Here, the left-hand side and G are as measured using cut-off L. As L→∞, we get the previous macroscopic value but we also now see the first quantum corrections.

### (d) Phase transition at L≈6 to the deep quantum gravity limit

**Figure 2.**
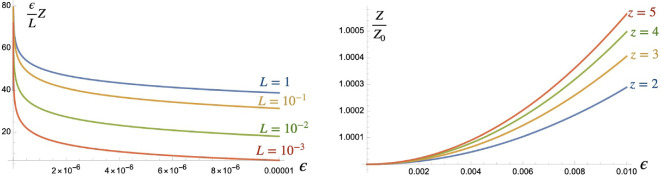
Numerical behaviour of Z for L≪6 as a function of ϵ and limit $Z\to Z_{0}$ for fixed $z=\frac{L}{\epsilon }>1$ as ϵ→0.

Revisiting figure 1a, we see that Z suddenly jumps to a very high value of order 1/ϵ as we decrease L below approximately L=6.4. For larger ϵ, the horizontal order 1/ϵ line moves down and the height of the ‘step’ at the critical value shrinks until there is no step at all above $\epsilon \approx 10^{-15}$ (for larger ϵ, Z transitions directly from the horizontal order 1/ϵ line on to the brown large L curve at the point where they meet). The phase transition is also visible in ⟨R⟩L in figure 1b, which suddenly jumps to a very high value that we will see is of order $1/\epsilon^{2}$ in the limit of small ϵ→0. The transition in the figure is at approximately L=7 and for larger ϵ this happens at slightly smaller L, down to 6.7 for $\epsilon =10^{-7}$ (for larger ϵ, the transition becomes significantly less pronounced and more gradual but is still two orders of magnitude starting at L=4.6 for $\epsilon =10^{-2}$). Note that Mathematica in this regime shows mild non-convergence warnings in the numerical integration so that the precise values here and as well as in figure 2 should be regarded as indicative. For example, the reported critical values of L are computed at machine precision 100 but the much lower default precision gives the Z transition for $\epsilon =10^{-20}$ at L=6.0 and that of ⟨R⟩L at L=6.6. Nevertheless, there is clearly a very different behaviour between the L-dominated regime for L≫6 studied above and the ϵ-dominated regime for L≪6, which we study in this section.

More precisely, below the critical value of L, one can see from figure 2 that Z is better approximated by something like,


$$Z∼c(\epsilon ,L)\frac{L}{\epsilon }\ln(\frac{L}{\epsilon }),$$

as ϵ→0, with some residual dependence in the coefficient c. To better understand this, we note that for the results in the small L phase being dominated by the limit ϵ→0, one can drop the exponential factor in the integrand, i.e.


$$Z\approx Z_{0}:=\int_{\epsilon }^{L}\prod_{i}d\lambda_{i}\;\frac{|(\lambda_{1}-\lambda_{2})(\lambda_{2}-\lambda_{3})(\lambda_{3}-\lambda_{1})|}{\lambda_{1}^{2}\lambda_{2}^{2}\lambda_{3}^{2}},$$

for L≪1. The ratio of the two, as we vary $z:=\frac{L}{\epsilon }$, is also shown in the figure. This $Z_{0}$ is independent of scaling the $\lambda_{i}$, so we can also view it as the original partition function (equation (3.2)) at G=∞ before we rescaled fields and with our current limits L,ϵ regarded as the physical $L^{phys},\epsilon^{phys}$ in this interpretation. Hence, this is the *deep quantum gravity* limit of the theory and is of interest in its own right.

Moreover, the $Z_{0}$ theory can be computed exactly as follows, and necessarily depends only on the ratio z. If $\lambda_{1}>\lambda_{2}$, then there are three regions for $\int d\lambda_{3}$ in relation to these, each of which can be solved analytically, which we then add together. If $\lambda_{2}>\lambda_{1}$, then we obtain the same as the other way around. This gives us


$$Z_{0}=\int_{\epsilon }^{L}\frac{d\lambda_{1}d\lambda_{2}}{\lambda_{1}^{2}\lambda_{2}^{2}}f(\lambda_{1},\lambda_{2}),$$

$$f(\lambda_{1},\lambda_{2})=|\lambda_{1}-\lambda_{2}|\left(L-\epsilon -4|\lambda_{1}-\lambda_{2}|+(\frac{1}{\epsilon }-\frac{1}{L})\lambda_{1}\lambda_{2}+(\lambda_{1}+\lambda_{2})(2|\ln(\frac{\lambda_{1}}{\lambda_{2}})|-\ln(\frac{L}{\epsilon })\right).$$

We then have two regions for $\int d\lambda_{2}$ in relation to $\lambda_{1}$, each of which can be solved analytically and the results added, to give a function of $\lambda_{1}$. This too can be integrated analytically, to give


$$Z_{0}=\frac{6}{z}\left(\ln(z)(z^{2}-1)+2z\;\ln(z)-4(z-1{)}^{2}\right).$$

**Figure 3.**
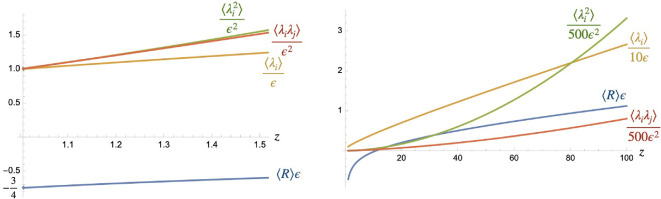
Expectation values in the deep quantum gravity $Z_{0}$ phase of the fuzzy sphere as a function of small and large z, respectively.


One similarly has exact expressions for expectation values in this phase, where we insert these observables into the integral for $Z_{0}$ and then divide by $Z_{0}$. These are, respectively,


$$\begin{array}{rl} \langle \lambda_{i}\rangle & =\frac{\epsilon (z-1)}{Z_{0}}\left(\left(1+4z+z^{2})\ln(z)-3(z^{2}-1\right)\right),\\ \langle \lambda_{i}^{2}\rangle & =\frac{\epsilon^{2}(z-1)}{9Z_{0}}\left(6(1+z)(1+5z+z^{2})\ln(z)-(z-1)(19+46z+19z^{2})\right),\\ \langle \lambda_{i}\lambda_{j}\rangle & =\frac{\epsilon^{2}(z-1)}{3Z_{0}}\left(6z(1+z)\ln(z)-(z-1)(1+10z+z^{2})\right),\\ \langle R\rangle & =\frac{\left(z-1\right)}{8\epsilon z^{2}Z_{0}}\left(6z(3+10z+3z^{2})\ln(z)-(z^{2}-1)(1+46z+z^{2})\right), \end{array}$$

for i≠j and are plotted in figure 3 as functions of z. We see a limiting value:

$$\langle \lambda_{i}\rangle =\epsilon ,\;\langle \lambda_{i}^{2}\rangle =\langle \lambda_{i}\lambda_{j}\rangle =\epsilon^{2},\;\langle R\rangle =-\frac{3}{4\epsilon },$$

as z→1, which has classical behaviour with zero variance of $\lambda_{i}$, and Ricci curvature that of a fuzzy sphere with metric $g_{ij}=\epsilon \delta_{ij}$. As z increases all, the expectation values increase, with the curvature crossing zero at z≈10.88. The asymptotic form for large z→∞ is by contrast


$$\langle \lambda_{i}\rangle ∼\frac{\epsilon z}{6},\;\langle \lambda_{i}^{2}\rangle ∼\frac{\epsilon^{2}z^{2}}{9},\;\langle \lambda_{i}\lambda_{j}\rangle ∼\frac{\epsilon^{2}z^{2}}{18(\ln(z)-4)},\;\langle R\rangle ∼\frac{z}{48\epsilon (\ln(z)-4)},$$

for i≠j, showing highly non-classical behaviour. This limiting form for large z implies, for the deep quantum gravity theory at G=∞ defined by $Z_{0}$ and fixed L that


$$\langle \lambda_{i}\rangle \to \frac{L}{6},\;\langle \lambda_{i}\lambda_{j}\rangle \to \frac{L^{2}}{9}\delta_{ij},\;\langle \lambda_{i}\lambda_{j}\rangle ∼\frac{L^{2}}{18\ln(\frac{1}{\epsilon })}\;(i\neq j),\;\langle R\rangle ∼\frac{L}{48\epsilon^{2}\ln(\frac{1}{\epsilon })},$$

as ϵ→0. From the two points of view, we also have


$$\frac{\Delta \lambda_{i}}{\langle \lambda_{i}\rangle }\to \sqrt{3},\;\frac{\langle \lambda_{i}\lambda_{i}\rangle }{\langle \lambda_{i}\rangle \langle \lambda_{j}\rangle }∼\frac{2}{\ln(z)-4}∼\frac{2}{\ln(\frac{1}{\epsilon })},\;\frac{\langle R\rangle }{\langle \lambda_{i}\rangle }∼\frac{1}{8\epsilon^{2}(\ln(z)-4)}∼\frac{1}{8\epsilon^{2}\ln(\frac{1}{\epsilon })},$$

for i≠j, which shows a fixed relative uncertainty in the quantum metric fields $\lambda_{i}$ similar to the deep quantum gravity limits of other known models. However, we also see $\left\langle \lambda_{1}\lambda_{2}\right\rangle$ going to zero and the Ricci curvature diverging as the regulator ϵ→0. This gives a flavour of this phase of the quantum gravity model as being fundamentally different from the large L phase studied before. In this phase, we would renormalize the original Z theory with G a function of ϵ or z rather than L.

### (e) Quantum gravity models on graphs

**Figure 4.**
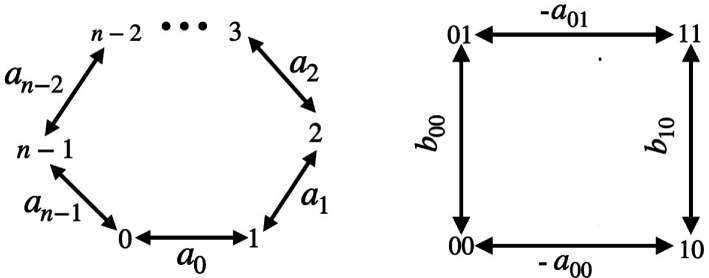
For the QRG of a graph, arrows are differential forms and a quantum metric is an assignment of a non-zero real ‘square length’ to each edge. The quantum metric for the n-gon defines a function a which at vertex i is $a_{i}$, and similarly for the square, two functions a,b with $a_{10}=a_{00},a_{11}=a_{01}$ and $b_{01}=b_{00},b_{11}=b_{10}$.

QRG can be applied to any algebra but it includes as a special case the algebra A of functions on a discrete set X. Here, possible $\Omega^{1}$ are in 1−1 correspondence with directed graphs with vertex set X. A vector space basis of 1-forms is $\left\{\omega_{x\to y}\right\}$ labelled by the arrows and $df=\sum_{x\to y}(f(y)-f(x))\omega_{x\to y}$ for f∈A encodes finite differences across each arrow. Multiplication of 1-forms by functions is $f\omega_{x\to y}=f(x)\omega_{x\to y}$ and $\omega_{x\to y}f=f(y)\omega_{x\to y}$, i.e. non-commutative even though the algebra A itself is commutative. In this way, a directed graph is literally an example of quantum geometry. A metric here, to have a bimodule inverse, has the form


$$g=\sum_{x\to y}g_{x\to y}\omega_{x\to y}⊗\omega_{y\to x},\;g_{x\to y}\in R_{\neq 0},$$

and only exists if every arrow has a reverse arrow, which we assume. In principle, the ‘metric square-length’ of an arrow could depend on the direction, but a natural notion of symmetry here is to suppose that it does not. We proceed in this edge-symmetric case, albeit this is not the only case of interest. Then a quantum metric is nothing other than an assignment of a non-zero real number or ‘square-length’ to every edge. Examples are shown in figure 4 for a Euclidean n-gon for n≥3 and a Lorentzian square, so called because the horizontal metric weights are assumed negative or ‘space-like’ (with $a_{00},a_{01}>0$) while the vertical ones are ‘time-like’ with $b_{00},b_{10}>0$). For the Euclidean case, the usual convention is to take all the $a_{i}>0$.

**Figure 5.**
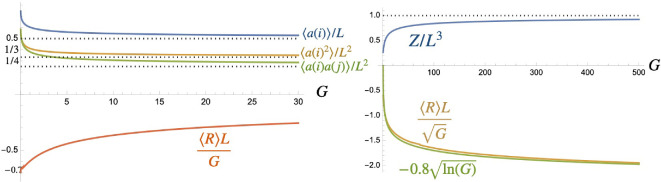
G-dependence of vacuum expectation values and partition function for quantum gravity on a triangle.


Proceeding in the n-gon case, there is a basis $e^{\pm }$ over the algebra of left-invariant 1-forms with respect to the group ${\mathbb{Z}}_{n}$, which we take to anticommute for the wedge product. Then the metric values regarded as a function a on the vertices provide the metric tensor in the form


$$g=ae^{+}⊗e^{-}+R_{-}(a)e^{-}⊗e^{+},$$

where $R_{\pm }(a)(i)=a(i\pm 1)$ is a shift mod n. There is a natural QLC that is unique for n≠4 (we stick to this one for all n) given in [18,21]. We omit the details and jump to the Ricci scalar:


$$R(i)=-\frac{1}{2}\left(\frac{a(i-1)}{a(i{)}^{2}}+\frac{a(i)}{a(i-1{)}^{2}}-\frac{1}{a(i+1)}-\frac{1}{a(i-2)}\right),$$

defined with respect to an obvious antisymmetric lift map i. We let the measure μ for integration be a itself as a natural choice. Then the quantum gravity partition function for n=3, say, is


$$Z=\int_{0}^{L}\int_{0}^{L}\int_{0}^{L}da(0)da(1)da(2)\;e^{\frac{1}{2G}\left(\frac{a(2)}{a(0)}+\frac{a(0)}{a(1)}+\frac{a(1)}{a(2)}-\frac{a(2{)}^{2}}{a(0{)}^{2}}-\frac{a(0{)}^{2}}{a(1{)}^{2}}-\frac{a(1{)}^{2}}{a(2{)}^{2}}\right)},$$

and similarly for general n. The action on closer inspection can be viewed as a scalar field action $\sum \rho \Delta_{{\mathbb{Z}}_{n}}\rho$ for the postive-valued function $\rho =R_{+}(a)/a$ and $\Delta_{{\mathbb{Z}}_{n}}$ the standard finite-difference Laplacian. One can quantize the theory via ρ or via the relative fluctuations b=a/A, where A is the geometric mean of the a(i), see [18]. Here, however, we just follow the straight theory. The results depend on G, which, unlike the previous model is dimensionless and cannot be scaled away. On the other hand, the dependence on L is exact because we could move to scale-invariant variables $\bar{a}=a/L$ and then compute the integrals independently of L. Figure 5 shows some plots; one has, e.g. for G=2,

$$\langle a(i)\rangle =0.234L,\;\langle a(i)a(j)\rangle =L^{2}\left\{ \begin{array}{cc} 0.403 & i\neq j\\ 0.435 & i=j \end{array}\right.,\;\frac{\Delta a(i)}{\left\langle a(i)\right\rangle }=\frac{\sqrt{\langle a(i{)}^{2}\rangle -\langle a(i)\rangle^{2}}}{\left\langle a(i)\right\rangle }=2.64,$$

which is similar to [18] and to which we now add the curvature


$$\langle R(i)\rangle =-\frac{0.318}{L}.$$

We see that the expectation values correspond to a regular polygon as the ground state, but with non-zero standard deviation or ‘quantum fuzziness’ and non-zero curvature as quantum gravity effects (the regular polygon having zero curvature in QRG). There is no need for a cut-off ϵ and these calculations do not noticeably change if we introduce a small ϵ.

The behaviour for G→0 can be analysed analytically as follows. The exponent in the integrand of Z is strictly negative other than zero at a(0)=a(1)=a(2). Hence, we introduce new variables a(0)=a,a(1)=a(1+x),a(2)=a(1+y) and when G is small, only x,y near to zero will contribute. Hence, we can replace the exponent effectively by a quadratic function of (x,y) and can then do the x,y integrations as Erf functions. Up to a constant, we obtain


$$Z∼G\int_{0}^{L}a^{2}da=\frac{GL^{3}}{3},$$

as G→0. Similarly with insertions of powers of a(0), and then by symmetry, we have


$$\langle a(i{)}^{m}\rangle \to \frac{3L^{m}}{m+3},\;\langle a(i)\rangle \to \frac{3L}{4},\;\frac{\Delta a(i)}{\langle a(i)\rangle }\to \frac{1}{\sqrt{15}},$$

for the triangle, which is consistent with the plots at G=0.01 (numerical integration at this value of G is already unreliable and we cannot say much more than this). This is not classical behaviour, but if we make the analogous change of variables for the n-gon then one similarly has


(3.5) $$\langle a(i{)}^{m}\rangle \to \frac{nL^{m}}{n+m},\;\frac{\Delta a(i)}{\langle a(i)\rangle }\to \frac{1}{\sqrt{n(n+2)}},$$

so that we will see classical behaviour in the limit of a circle at large n. In addition, for the curvature of the triangle, we have


$$\langle R(i)\rangle ∼-0.699\frac{G}{L},$$

verified numerically for G=0.01 and consistent with G→0 asymptotic estimates for all n along the same lines as above. This again fits the weak gravity limit where the regular n-gon has zero curvature.

At the other extreme, i.e. for the strong gravity limit, we have


$$Z\to L^{3},\;\langle a(i)\rangle \to \frac{L}{2},\;\langle a(i)a(j)\rangle \to L^{2}\left\{ \begin{array}{ll} 1/4 & i\neq j\\ 1/3 & i=j \end{array}\right.,\;\frac{\Delta a(i)}{\langle a(i)\rangle }\to \frac{1}{\sqrt{3}},$$

as G→∞. These limits are the same as working in the theory where G=∞ and we just drop the exponential in the integrand, i.e. work with the rather trivial


$$Z_{0}=\int_{\epsilon }^{L}\prod_{i}da(i)=(L-\epsilon {)}^{n}=L^{n}(1-\frac{1}{z}{)}^{n},\;z:=\frac{L}{\epsilon },$$

which is, moreover, the same for any graph with n edges (it does not need to be an n-gon). Clearly, for powers of the metric other than $m_{i}=-1$,


$$\langle a(0{)}^{m_{0}}\cdots a(n-1{)}^{m_{n-1}}\rangle =\prod_{i}\left(L^{m_{i}}\frac{[m_{i}+1{]}_{\frac{1}{z}}}{m_{i}+1}\right);\;[m{]}_{\frac{1}{z}}:=\frac{1-\frac{1}{z^{m}}}{1-\frac{1}{z}},$$

in which, for positive powers as above, we can just take ϵ=0 or z=∞. Here, expectation values are given by inserting the observable into the integral for $Z_{0}$ (i.e. integrating it) and dividing by $Z_{0}$.

The curvature, however, does depend on the graph and in the $Z_{0}$ theory for the n-gon, we do need the regulator ϵ to be able to do the integrals. The result, independently of n, is


$$\langle R(i)\rangle =-\frac{1}{L}\left(\frac{z+1}{2}+\frac{\ln(\frac{1}{z})}{1-\frac{1}{z}}\right)∼-\frac{1}{2\epsilon },$$

if we expand for large z. This now depends on how we take the joint limit ϵ→0 as G→∞. For example, setting $\epsilon =L/(4\sqrt{G})$ would give $\langle R(i)\rangle L/\sqrt{G}∼-2$, while another choice gives a better fit to


$$\langle R(i)\rangle L∼-0.8\sqrt{G\ln(G)},$$

as shown in the figure (this is a good fit until approximately $G=10^{4}$, the actual formula is likely to have several terms as we saw in the fuzzy sphere deep quantum gravity phase). This gives a flavour of how the divergence of ⟨R(i)⟩ as G→∞ can be modelled in the limit $Z_{0}$ theory. We do not see a phase transition as for the fuzzy sphere but, by contrast, a smooth transition in the above between weak and strong gravity limits.

We do not give any details for the Lorentzian square because a new treatment extending [20] will be given elsewhere [27]. Suffice to say that we see this as the group ${\mathbb{Z}}_{2}\times {\mathbb{Z}}_{2}$ (and numbered the vertices accordingly), which leads to a different $\Omega^{2}$ from the case of the 4-gon, as the natural left-invariant basis of 1-forms are different. This time, there is a moduli of QLCs with an angle parameter θ which does not, however, enter the Einstein–Hilbert action,


$$S_{g}=(a_{00}-a_{01}{)}^{2}(\frac{1}{a_{00}}+\frac{1}{a_{01}})-(b_{00}-b_{10}{)}^{2}(\frac{1}{b_{00}}+\frac{1}{b_{10}}),$$

obtained from the Ricci scalar curvature R and measure μ=ab built from the metric. We again cut off metric values at scale L and have non-zero ⟨a⟩ and Δa/⟨a⟩ for the metric and a non-zero limit for these as G→∞, much as in our models above. Moreover, for the Ricci scalar at the different vertices,


$$\langle R(00)\rangle =\langle R(11)\rangle =\frac{9ıG}{8L^{2}}(1+\cos(\theta )),\;\langle R(01)\rangle =\langle R(10)\rangle =\frac{9ıG}{8L^{2}}(1-\cos(\theta )),$$

which is imaginary. The coupling constant G as in equation (3.1) for this model has dimensions of length^2^, the same as L. Hence $\bar{G}=G/L$ is comparable to the dimensionless G for the polygon, after which we see that the result is analogous to the weak gravity limit there.

## 4. How elementary particles could emerge from quantum gravity

It is an old idea of Kaluza and Klein that gravity on a product spacetime M×K, where K is a compact Riemannian manifold with isometry group G, includes as certain modes within it Yang–Mills gauge theory on M with gauge group G and gravity on M, see [28] for a review. In addition, a scalar field on M×K appears as an infinite tower of fields with different masses coupled to the gauge field. This old idea raised the hope of thinking of gauge fields as just part of a higher-dimensional gravity theory but did not work too well in practice. For one thing, the metric on the product has to be very special, that is, with coefficients that are constant on K in the Killing form basis (the ‘cylinder ansatz’), i.e. this is a rather special ansatz and a way of looking at things but lacks explanatory power. Moreover, when $K=S^{1}$ and we want to recover electromagnetism, the size of this circle fibre comes out as 23 $\lambda_{P}$. Similarly for the weak force, we would need 11 $\lambda_{P}$. In other words, we would need a compact fibre so ‘curled up’ as to be significantly affected by quantum gravity effects. This in turn throws up the idea that the particular structure of forces observed in Nature could come out of quantum gravity.

In addition, Connes and Chamsedine [11] looked at replacing K by a (finite) non-commutative geometry $A_{f}$. Their idea was that if functions on spacetime, $C^{\infty }(M)$, are replaced by $C^{\infty }(M)⊗A_{f}$ then a particular ‘spectral triple’ or abstract Dirac operator on this product could be used to encode the matter content of the Standard Model of elementary particles. This again was a particular product ansatz but did produce a postdiction for the Higgs mass and a novel mass relation among some matter fields. The Dirac operator governs fermionic matter fields, while gauge fields in this approach enter as fluctuations of the Dirac operator.

In recent work [29–31], we have begun to put these two ideas together, i.e. to revisit Kaluza–Klein theory with a non-commutative fibre as a quantum gravity effect. This could then explain the further structure of particle physics down the line, once Dirac operators on the product have been analysed. What one finds notably in [31] is that when $A_{f}$ is the fuzzy sphere, everything now conspires to perfectly express the *full content* of gravity on $C^{\infty }(M)⊗A_{f}$ as exactly:

(i) gravity on M with its Einstein–Hilbert action for a field $\tilde{g}$.
(ii) $SU_{2}$ Yang–Mills gauge theory on M for a field $\tilde{A}$ but slightly generalized in that $||\tilde{F}|{|}^{2}$ uses a field $h_{ij}$ to contract the $su_{2}$ indices of the curvature $\tilde{F}$ of $\tilde{A}$.
(iii) $h_{ij}$ as a positive-matrix-valued Liouville field on M which at each point is the metric $h=h_{ij}s^{i}⊗s^{j}$ on the fuzzy sphere at that point. Its Liouville potential is the Ricci scalar of h on the fuzzy sphere.

Specifically, the quantum metric on $C^{\infty }(M)⊗A_{f}$ is *forced* in local coordinates $x^{\mu }$ on M to have the general form [29]:


$$g=g_{\mu \nu }dx^{\mu }⊗dx^{\nu }+A_{\mu i}(dx^{\mu }⊗s^{i}+s^{i}⊗dx^{\mu })+h_{ij}s^{i}⊗s^{j},$$

and [31] proves that there is then a unique QLC, computes its Ricci scalar and then identifies its integral as the action for certain fields ${\tilde{g}}_{\mu \nu },{\tilde{A}}_{\mu i}$ built from $g_{\mu \nu },A_{\mu i},h_{ij}$ above. Here, integration on the fuzzy sphere is taken to be the map that sends an element to its spin 0 component in the expansion of $A_{f}$ into $SU_{2}$ representations according to its action as orbital angular momentum on the fuzzy sphere.

Now, to get Yang–Mills fields in [31], one has to assume that $h_{ij}=h\delta_{ij}$ (the round metric on the fuzzy sphere) for a constant h. One also has similar problems in the original Kaluza–Klein theory. So why should this hold? Our tentative answer is that we need to proceed to quantize the $h_{ij}$ metric on the fuzzy sphere and then use its expectation value $\left\langle h_{ij}\right\rangle$ to understand what the model looks like on M. In this case, we saw in §3 that in quantum gravity on the fuzzy sphere, the expected value of the metric is indeed the round metric $\langle h_{ij}\rangle =h\delta_{ij}$ (since all the $\lambda_{i}$ have the same expectation value independently of i) and, moreover, we saw in §3b that in the large L phase, $h=\langle \lambda_{i}\rangle \approx L$. On the other hand, this might be expected to be a constant if we regularize and renormalize uniformly over spacetime. So, this could potentially provide an explanation for what we observe at low energies on M. Also note that for the weak force, we need $h=L=11^{2}=121$ in Planck area units, hence well in the L≫6 phase. But if such ideas are applied to the strong force (which is not immediately the case due to a different gauge group), this is approximately 20 times stronger (this is dependent on the energy scale, but a typical strong fine structure constant value is $\alpha_{S}\approx 0.7$, compared to $\alpha_{W}\approx 1/30$ used for the weak force calculation in [31]). This translates to L≈6, so we could enter the other phase of the theory. This would need to be explored for more relevant quantum geometries (with $SU_{3}$ symmetry) as fibre.

Also looked at in [29–31] is how scalar matter fields on the product look in terms of M. The big difference from the classical Kaluza–Klein theory with compact fibre K is that when $A_{f}$ is finite-dimensional, a single field on the product appears as a finite multiplet of fields on M, with different masses according to different eigenvalues for the Laplacian on the fibre. The holy grail here would be to solve the ‘generations problem’, that is, to explain why the electron, its neutrino and the up/down quarks appear to be repeated two more times with particles of different mass but otherwise are identical. For this, we would first need to extend the analysis to fermionic fields and the moduli of Dirac operators on the product within QRG, another direction for further work.

## 5. Quantum geodesics

If quantum spacetimes have no points, then it is hard to imagine that they have geodesics. These can nevertheless be addressed by taking a fluid-mechanics-like point of view. Thus, consider not one particle but a dust of particles with density ρ on a (pseudo) Riemannian manifold M, where each particle moves on a geodesic. At first sight, one might think that the tangent vectors of all these particles fit together to form a vector field X that also evolves in time. This will need to obey the *geodesic velocity equation:*


$$\dot{X}+\nabla_{X}X=0,$$

where the dot means derivative with respect to the geodesic ‘time’ parameter, which we will denote by s to avoid confusion in the case where M is spacetime. Moreover,


$$\dot{\rho }=-X(d\rho )+div(X)\rho ,$$

is the continuity equation in the fluids case, but we will refer to it as the *density flow* equation. It turns out, however, that X cannot be determined by ρ, but in addition the geodesic velocity equation can be solved without reference to any particles at all! Hence, if we have access only to densities and not particles themselves, we have to rip apart the usual concept of a geodesic and re-assemble it in reverse order: we think of the geodesic vector field X as a physical quantity in its own right, and then after solving for X we can evolve any initial density to later time via the density flow equation. This new field X captures a lot of geometry, for example [24]:


$$\frac{D}{Ds}div(X)=-||\nabla X|{|}^{2}-Ricci(X,X),$$

where $\frac{D}{Ds}$ is the convective or co-moving derivative. This directly shows the role of the Ricci tensor in controlling how a fluid expands or contracts in free-fall.

Next, we go one stage further and take a leaf out of the quantum mechanics book, replacing ρ by a complex amplitude ψ with $\rho =|\psi {|}^{2}$. We replace the density flow by the *amplitude flow:*


$$\dot{\psi }+X(d\psi )+\frac{1}{2}\psi div(X)=0.$$

This is equivalent for ψ real and positive, but for other, e.g. complex, values it opens up the possibility of interference phenomena that remain to be explored, for example around a regular classical black hole. The ‘quantum mechanical interpretation’ here applies to the observer with time s, which is external to the system. In General Relativity, geodesics entail a so-called ‘God’s eye view’ and that eye is now being upgraded to a quantum mechanical language.

**Figure 6.**
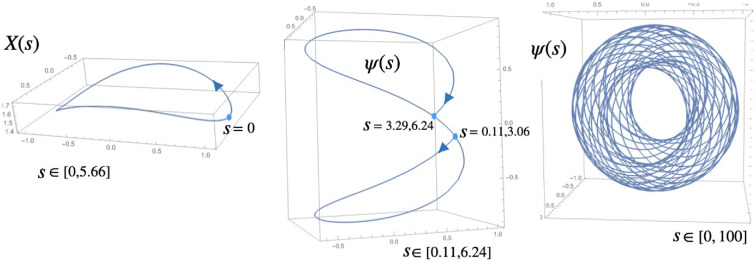
Quantum geodesic on the fuzzy sphere. We restricted to wave functions of the form $\psi =\psi_{i}x^{i}$ with $\psi_{i}$ real. Unitary evolution ensures $\vec{\psi }$ stays on a sphere, but there are two disks into which the geodesic never enters. Image adapted from [24].


At this point, everything extends to a general QRG. Given $\left(A,\Omega^{1},d\right)$*,* we let $X\in \chi :={,}_{A}\mathrm{hom}(\Omega^{1},A)$ be a left vector field (a left-module map for the action of A from the left). A QLC on $\Omega^{1}$ induces a right-handed connection $\nabla_{\chi }:\chi \to \chi {⊗}_{A}\Omega^{1}$ and the geodesic velocity equation in the simplest form becomes [23]


$$\dot{X}+\frac{1}{2}[X,div(X)]+(id⊗X)\nabla_{\chi }X=0,$$

where div(X) is the divergence of X defined ideally via the QLC. In the simplest case, we let ψ∈A (or rather in a completion of this where $\int \psi^{*}\psi <\infty$ with respect to a chosen integration) and take the same equation as above extended to this, being careful about the order. This works in the simplest case where ∫ is a twisted trace and ∫div(X)=0 for all X. We refer to [24] for details. Under these assumptions, one can show that,


$$\frac{d}{ds}\int \psi^{*}\psi =0,$$

so that we can in principle normalize $\rho =\psi^{*}\psi$ to ∫ρ=1. Here, ρ is an evolving positive operator, not necessarily a real density, but one still has a probabilistic picture with expectation value:


$$\langle a\rangle =\frac{\int \psi^{*}a\psi }{\int \psi^{*}\psi },$$

for observable a∈A in state ψ. An example of a quantum geodesic on the fuzzy sphere with metric $g_{ij}=\mathrm{diag}(4,3,1)$ is given in figure 6 taken from [24], where X is solved in terms of elliptic Jacobi functions,

$$X^{1}(s)=-\frac{1}{\sqrt{2}}\;\text{sn}(s|-\frac{1}{2}),\;X^{2}=\text{cn}(s|-\frac{1}{2}),\;X^{3}=\sqrt{2+{\text{sn}}^{2}(s|-\frac{1}{2})}.$$

Here, $X=f_{i}X^{i}$ with $f_{i}$ a dual basis to the $s^{i}$ in §3. This is an easy case where each $X_{i}$ is constant on the fuzzy sphere, and similarly ψ is solved in a easy case where it is linear in the coordinates.

This machinery has the power to give coordinate-free predictions in quantum spacetime models. So far, this has only been explored in first approximation (without consideration of the functional analysis), but two take-aways from [25] for the spacetime (1.1) appear to be:

(i) An initial ψ which is a Gaussian bump at a point in spacetime and which then evolves (approximating the worldline of a point particle) and gets quantum corrections that are inverse to the Gaussian width, i.e. a perfect point source appears to be forbidden by quantum gravity due to infinite corrections mediated by the quantum spacetime.
(ii) An initially real-valued ψ typically gets complex quantum corrections, i.e. does not stay real. Hence quantum gravity will lead to the kind of interference effects mentioned above.This new technology requires further study and in particular could be applied to non-commutative black hole models and expanding universe Friedmann–Lemaitre–Robertson–Walker cosmological models. Models of these within QRG appeared in [32] and one interesting feature is a *dimension jump* where, if we replace the sphere at each r,t by a fuzzy sphere and look for spherically symmetric static solutions with r,t classical, we end up with a metric similar to the five-dimensional Tangherlini black hole. We can also replace the sphere at each point by ${\mathbb{Z}}_{n}$ or its limit of a circle with its limiting two-dimensional calculus. The same goes for cosmological models, an expanding $S^{3}$ at each time can be replaced by an expanding fuzzy sphere with the same Friedmann expansion equation as the Standard Cosmological Model. Dispersion relations have been long-conjectured to be modified in quantum spacetime but these could now be determined by looking at quantum geodesic flows.

Finally, quantum geodesics should also provide a new framework to see particle creation or Bekenstein–Hawking radiation. This has already been demonstrated on the integer line graph (a 1-dimensional lattice) in [21] as follows. We assume a constant flat metric a(i) at far left (large negative i) and again at far right (large positive i) and a varying metric in a region around i=0. Now, we can solve the wave equation $(-\Delta +m^{2})\phi =0$ starting with boundary conditions matching a plane wave and its conjugate at far left for the metric there and then solving the recursion entailed in the wave equation to extend ϕ to all i. At large i we match ϕ to a plane wave and its conjugate for the metric there. The coefficients of the plane waves are then promoted to annihilation and creation operators in quantum mechanics and used to compute the expectation value of the occupation number operator for the far right theory in the vacuum state of the far left theory. A question for the future is whether we can see similar effects with quantum geodesics. Here, one approach is to follow [23] where the ‘Klein–Gordon flow’ $\dot{\psi }=\frac{ı\hbar }{2m}\Delta \psi$ can be viewed as an amplitude flow for a certain quantum geodesic velocity field defined with respect to a generalized (not symmetric) quantum metric on the relevant Heisenberg algebra. Underlying the latter here was ordinary Minkowski spacetime with a background Maxwell field, but the ideas could be adapted to any QRG.

## 6. Concluding remarks

The quantum spacetime hypothesis, which we explored, is agnostic as to the ‘true’ theory of quantum gravity, in that several different approaches ranging from string theory to loop quantum gravity and spin networks suggest that this is closer to the real world than a continuum due to quantum gravity effects. The logical corollary of this, however, is that it would be more consistent to construct quantum gravity on a quantum spacetime in the first place, preferably the same one that then comes out as an effective description. In this respect, approaches built on a continuum such as supergravity and string theory, while very interesting, do not directly address the problem at hand of spacetime not being a continuum. Among non-continuum starting points, QRG links with lattice gauge theory and how it relates to spin networks are studied in [33].

By contrast, letting the coordinate algebra be non-commutative and/or finite dimensional, as seen in our baby quantum gravity models, turns the infinities that plague straight continuum quantum gravity into ordinary divergences that are more reasonable to handle, even if we still need to renormalize. We now return to our check-list (1)–(5) and ask what we have learned from such models and the related formalism of QRG so far. For (1), we have seen some small hints in §5 that point-sources are not physical due to quantum corrections. This needs to be looked at further and in addition is sidestepped for graphs, where the entire space is discretized so that the issue does not arise. For (5), we noted in §4 a mechanism for how multiplets of particles transforming under a local gauge symmetry could naturally arise from gravity on a product *provided* we take the fibre to be a QRG. Taking A to be highly non-commutative in the sense of trivial centre was key here for the mechanism. For (3), baby quantum gravity models in principle allow one to ‘count’ everything explicitly and there is no particular problem doing the computations. Moreover, optimal transport theory on graphs takes a similarly probabilistic approach but has well-developed links with entropy and quantum information [34]. We also know from QRG models that thermalization of the vacuum can be seen as a general feature of transition through a region of varying metric [21] and how one can do calculations. Hence, this question appears on track but requires more development. Point (4) relates to the nature of quantum measurement and at the moment here, rather than solving anything, we may need a paradigm shift. Quantum geodesics, which apply quantum methods to the process of observing geodesic flow, could be a step in the right direction.

Finally, for (2), we do have indications that dark energy or the cosmological constant could arise from quantum spacetime. First, we saw in the baby quantum gravity models a non-zero relative uncertainty in the metric, which suggests some kind of vacuum energy. For a discrete circle modelled by ${\mathbb{Z}}_{n}$, we saw in equation (3.5) that this goes as $\frac{1}{n}$ or the relative variance $\frac{1}{n^{2}}$. One might speculate that the energy density corresponding to this should be the Planck density (due this being a quantum gravity effect) times the relative variance. We have no strong argument for this, but note that if we crudely model the universe as a discrete circle ${\mathbb{Z}}_{n}$ then we might want $n=5.5\times 10^{61}$ as the size of the Universe in Planck units, and in this case


$$\frac{Planck\;density}{n^{2}}\approx \frac{5.1\times 10^{93}}{(5.5\times 10^{61}{)}^{2}}\approx 2\times 10^{-29}{\;g\;cm}^{-3},$$

compares very well with the observed density of the Universe of $9\times 10^{-30}$, most of which is believed to be dark energy. Or from another angle, for a finite chain ∙–∙-⋯ - ∙ with n nodes, one finds [35] that the natural QRG is q-deformed with $q=e^{\frac{ı\pi }{n+1}}$ compared to an infinite lattice. But in Euclideanised 2 + 1 quantum gravity, q-deformation corresponds to introduction of a cosmological constant $\Lambda =\lambda_{c}^{-2}$ via $q=e^{\frac{\lambda_{c}}{\lambda_{P}}}$. Equating these (without the ı) with our above value of n gives $\lambda_{c}∼\lambda_{P}\frac{n}{\pi }∼3\times 10^{26}$m or a cosmological constant $10^{-53}$m^-2^, compared to the observed value of $10^{-52}$m^-2^. Thus, while these arguments are rough and speculative, they could be seen as supporting the idea that the cosmological constant arises from the non-commutative/discrete nature of spacetime. Note that from the point of view of 2 + 1 quantum gravity, the introduction of the cosmological constant suggests a q-deformation of equation (1.2) to the quantum group $U_{q}(su_{2})$ as the relevant model quantum spacetime [10]. This means that the curved $SU_{2}$ momentum space dual to equation (1.2) becomes the quantum group $C_{q}(SU_{2})$, i.e. the cosmological constant from this point of view leads to non-commutative momentum space, in addition to changing the spacetime further.

To proceed from such hints to actual calculations, a better measure of the energy density in the gravitational field is $\frac{R}{16\pi G_{N}}$ where $G_{N}$ is the actual Newton constant (in our baby models, G had a similar role but was of whatever dimension was needed for a dimensionless exponent, for example of dimensions length${,}^{2}$ for the fuzzy sphere). We are interested in quantum-gravity-induced curvature corrections and for the fuzzy sphere our next-to-leading order result in equation (3.4) can be written as $2\langle R\rangle /L^{2}$. If we match the curvature there with our observed curvature of order $1/\lambda_{c}^{2}=\Lambda$ then the quantum-gravity induced energy density is


$$\frac{\Lambda c^{2}}{8\pi G_{N}L^{2}}\approx \frac{10^{-52}c^{2}}{1.7\times 10^{-12}L^{2}}\approx \frac{5.3\times 10^{-30}}{L^{2}}{\;g\;cm}^{-3},$$

where c is the speed of light and the final expression is converted from cubic metres to cubic cm. Since we assumed that L≫6, this energy density is even smaller than we wanted but provides a proof of concept as to how this might work. The problem, however, is that the dimensionless parameter L enters here as a regulator and its physical interpretation is not fully understood. We could, for example, replace it to leading order by $L=\langle \lambda_{i}\rangle =\langle \lambda_{i}^{phys}\rangle /G=\lambda_{U}^{2}/G$ for $\lambda_{U}$ the size of the Universe, but the question would still be the value of G at this scale. The obvious choice $G=\lambda_{P}^{2}$ leads to an extremely small answer and we would need something rather closer to the other end $G=\lambda_{U}^{2}$. Alternatively, we could be in the L≪6 phase of the model and in this case have to contend with a divergence of ⟨R⟩ in terms of a regulator ϵ and its more significant renormalization. There is only so much one can learn about the real world by analogy with our baby models, but this gives a flavour of some issues for further work towards more appropriate models.

Turning now to the mathematics, the integration measure μ in constructing the baby quantum gravity models was chosen somewhat to taste and a more systematic approach to this is needed. Some results in this direction are suggested by compatibility of the integral with divergence as in the theory of quantum geodesics [24]. Different choices will change the dimension of G and the flavour of the model. We will also need to understand better and perhaps modify the construction of the Ricci tensor. Both issues will need to be informed by a theory of variational calculus in non-commutative geometry, which is currently lacking and which is needed to connect the path integral approach adopted so far, to an operator-level ‘Hamiltonian’ approach. Variational calculus in turn needs either a proper understanding of the Hopf algebroid of differential operators on a differential algebra or, on the dual side, of its jet bundle. Both of these are an active area of development at the time of writing, with some initial work already done [36–38]. A further clue is that quantum geodesics themselves should be understood in terms of variational calculus as is the case classically.

## Data Availability

This article has no additional data.
